# Parents of Children and Young People With Long‐Term Physical Health Conditions—Experiences of Navigating School

**DOI:** 10.1111/cch.70132

**Published:** 2025-07-31

**Authors:** Vicky Hopwood, Simon Pini, Megan Roker

**Affiliations:** ^1^ Leeds Institute of Health Sciences University of Leeds Leeds UK

**Keywords:** advocacy, children and young people, disability, education, inequalities, long‐term physical health conditions, needs, parents

## Abstract

**Background:**

Children and young people (CYP) with different long‐term physical health conditions report common needs at school, but little is known about the views of their parents. This research sought to provide the parent perspective on what secondary school‐aged CYP with medical conditions require at school and the role parents play in negotiating support for their children.

**Methods:**

Parents of CYP aged 11–18 years attending school in the United Kingdom, with one of 10 long‐term physical health conditions, took part in interviews about their children's school experiences. To prioritise parent voice, participants completed a preparation activity to encourage them to have more control over the interviews. A needs analysis from the previous INSCHOOL CYP project was used as the basis for a framework analysis of parent interviews and supplemented with an analysis workshop with three parents.

**Results:**

Twenty‐seven parents participated from September 2023 to May 2024. Parent views of the needs their CYP have at school corroborated the six needs previously identified by CYP themselves: to safely manage health at school; for a flexible education pathway; to be acknowledged and listened to; to be included in and supported by the school community; to build towards the future; to develop attitudes and approaches to coping in school. In addition, parents reported far more examples of their CYP having significant emotional and mental health needs. Parents played a crucial role in compensating for unmet needs, advocating for CYP, advising schools and championing equality and inclusion. Parents also had their own needs: to feel confident their CYP are safe at school; to be listened to and involved; to have information about rights and responsibilities; and to have mental health and emotional support.

**Conclusion:**

This parent‐focused study strengthens an existing needs analysis for CYP, adding to evidence showing significant unmet needs in school. Parents play a crucial role in addressing failures to meet these needs. Navigating the system to secure support can have negative implications for home‐school relationships and parent well‐being. Requirements for parental agency to ‘battle’ through health and education systems exacerbate health inequalities, as not all parents are able to fulfil this function. Improvements are needed in the support currently offered to CYP with health conditions and their parents.

## Summary

- Parents of children with long‐term physical health conditions confirm previous research findings from young people that children have six common needs at school. These needs are to (1) safely manage health at school, (2) provide a flexible education pathway, (3) acknowledge and listen in the right way, (4) be included and supported by the school community, (5) build towards the future and (6) develop attitudes and approaches to cope in school and have good mental health. These needs are present across children with a broad spectrum of chronic health conditions and are often unmet at school.
- Parents play a vital role in securing support to meet unmet needs at school, advocating and compensating for children—often in their own time and out of their own pockets.
- Mental health support for children emerged as a key area to help children cope with their health condition or redress effects of unmet needs. Parents also require emotional support.
- Parents ‘battle’ and ‘fight’ for support for children's needs to be met, which can lead to health and education inequalities and negative child/parent/school relationships.
- Parents want to feel confident that children with medical conditions are safe, listened to and included at school. To better address unmet needs, school and healthcare professionals could provide a named contact who has knowledge of medical conditions and how to support them. Professionals should better provide information on the rights of CYP with medical conditions and the roles of respective authorities to address these. Government guidance to support children with medical conditions in England has not been updated since 2015 and would benefit from review.

## 1 Introduction

Long‐term physical health conditions (LTCs) require management or treatment over several years and can be controlled, but not cured, with medication and therapies (Busse et al. 2010; NHS 2023). Definitions of ‘long‐term physical’ or ‘chronic health’ vary (Spencer et al. 2022) and can include conditions as diverse as skin conditions, asthma, diabetes and cancer. Estimates suggest there are around 1–1.7 million children and young people (CYP) in the United Kingdom with a LTC (All Parliamentary Group for Diabetes 2018; Hagell et al. 2013; Lewis and Lenehan 2014; National Institute for Health and Care Excellence 2023). This equates to approximately 11%–17% of UK pupils (Gov UK 2023; Hopwood et al. 2024; National Institute for Health and Care Excellence 2023).

Research has shown that school‐age children with LTCs can suffer health and education inequalities (Hopwood et al. 2024; Mon Williams et al. 2023). LTCs have been shown to negatively impact attendance and academic attainment (Eloi et al. 2019; Forrest et al. 2013; Hopwood et al. 2024; Jay et al. 2023; Spencer et al. 2023a). They can also be detrimental to fostering a sense of school belonging, peer relationships and inclusion in school activities (Bryan et al. 2021; Hopwood et al. 2024; Spencer et al. 2023a).

Despite reported deficits, direct accounts of CYP's educational experiences from those living with LTCs were relatively limited until recently. Capturing lived experience is important given discourse suggesting that collaborating with intended recipients of support leads to more valuable results and better designed services (Lea et al. 2018; Spencer et al. 2023a; Taylor et al. 2018; Van Schelven et al. 2021). The INSCHOOL project addressed this gap by adopting a participant‐driven methodology to capture empirical first‐hand accounts of school life from CYP across a diversity of LTCs (Hopwood et al. 2024; Spencer et al. 2022, 2023a, 2023b).

**FIGURE 1:**
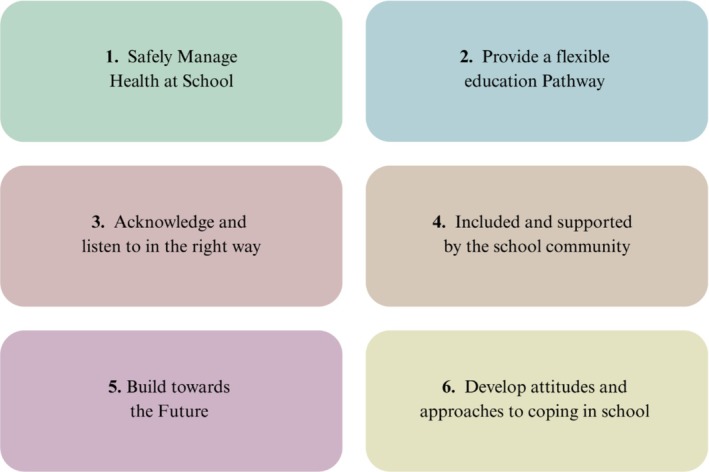
Six common needs of CYP with LTCs in school. *Note:* CYP = children and young people.

INSCHOOL identified six unmet needs common across secondary‐aged CYP with LTCs in relation to school life (see Figure 1 based on findings from Spencer et al. 2023a).

These wide‐ranging needs support evidence and calls for the experiences of CYP with a LTC to be viewed in a holistic manner to tackle inequalities faced by this population (Mon Williams et al. 2023).

It is essential to elicit and value the patient voice when trying to understand experiences of those with LTCs, but ‘when the patient is a child, this includes understanding the views and experiences of their parents’ (Smith and Firth 2011, 2). This is important, considering the valuable role parents play in identifying and mediating the needs of CYP with LTCs at school (Ickmans et al. 2022) and which include assisting children to self‐manage their health condition, participate in activities and to build a sense of belonging and relationships with peers (Lin and Lee 2024).

The UK Children and Families Act (2014) placed a duty on schools to provide support for pupils with medical conditions. Accompanying guidance provided suggestions on how schools can support pupils (Department for Education 2014, 2015).

Research with parents, most notably in relation to children with cancer, has exposed limitations in the education system. These include a lack of support to help CYP to catch up and keep up with school work, the absence of an education intermediary to promote children's needs at school, limited communication between the family, health and education services and limited application of statutory guidance (Bryan et al. 2021; Delloso et al. 2021; Lum et al. 2017; Pini, Hugh‐Jones, et al. 2011). Evidence from other LTCs, such as chronic pain, shows different parenting behaviours can influence CYP's autonomy, resilience and decision‐making (Evans et al. 2010; Ickmans et al. 2022; Palermo et al. 2014), which can impact condition management.

Parents usually act as agents for support and conduits for health information (Bryan et al. 2021). This requires knowledge of the health condition and educational system and can have practical implications such as a need to take time off work (Palermo et al. 2014). Parents can be seen as natural advocates for children (McCammon et al. 2018; Smith‐Young et al. 2022), and this activism has been suggested as a coping strategy for parents of children with disabilities and LTCs (Ewles et al. 2014; Smith et al. 2022; Smith‐Young et al. 2022). Parents have been shown to display strong emotions when acquiring the right support and services for children, with some adopting ‘aggressive tactics’ to ensure needs are met (Smith‐Young et al. 2022).

The relative ability and opportunity for parents to advocate for CYP impacts on the likelihood of children facing disparities in service provision, with more inequalities being faced by CYP with parents less able to advocate for them (Lee et al. 2021). Barriers to parental advocacy include time commitment, lack of knowledge and support from professionals and perceived stigma surrounding health conditions (Smith‐Young et al. 2022).

Statutory guidance in the United Kingdom on supporting pupils at school with medical conditions contains limited information on the parent role, other than to inform the school and to contribute to developing an Individual Health Plan, which is a plan to set out CYP's medical needs and care at school (Department for Education 2015).

Whilst there is evidence from specific conditions on the role of parents, further research is required across a range of health conditions. This is because although there are some issues that are specific to conditions, the INSCHOOL project has shown that there are many commonalities in fundamental needs. To encourage a more complete understanding of the needs of CYP with LTCs in school, it is important to investigate parental experiences of the school system and how these may enhance information gathered directly from CYP. This study will follow on from our existing INSCHOOL project to add the parent's perspective.

## 2 Present Study Research Questions

This study aims to add depth and context to the school experiences described by CYP aged 11–18 years in the INSCHOOL project (Spencer et al. 2023a) by investigating:

1. What do parents of secondary school age CYP with LTCs say about their child's experiences of school?
2. What role do parents play in navigating this experience?

## 3 Methods

### 3.1 Design

This study was underpinned by a pragmatic epistemology (Cornish and Gillespie 2009) to maximise opportunities for parents to participate and use the most appropriate methods for the situation, rather than being theory driven. The study was cross‐sectional and employed participant‐led qualitative interviews for data collection (Vaughn and Jacquez 2020). Participatory research seeks to collaborate and co‐construct research with those affected by the issue being studied (Vaughn and Jacquez 2020).

Three parents with lived experience of having a child with a LTC helped inform the study, in keeping with well‐documented benefits of this approach (Taylor et al. 2018; Van Schelven et al. 2021). They were recruited through clinicians and researcher networks and subsequently consulted on design, question priorities and study documentation as well as taking part in pilot interviews. One parent also participated in the main study.

Additionally, four parents who completed study interviews took part in a coanalysis workshop to interpret and verify the findings. This was to address evidence gaps suggesting the analysis stage seldom involves participants (Spencer et al. 2023b). Both researchers and parents reflected that involving parents in design and analysis was a good investment and helped to better capture and shape the presentation of their stories.

### 3.2 Sampling Strategy and Recruitment

The intention was to sample parents of mainstream secondary school aged children (11–18 years) with a range of LTCs. This was to tackle reported knowledge gaps across CYP with different LTCs and known risk factors such as severity, duration and visibility (Pless and Nolan 1991) and to ensure the school age range was consistent with the INSCHOOL study. A mix of convenience and purposive stratified sampling was planned to recruit for diversity of parental roles and CYP characteristics. The aim was to include a mix of mothers and fathers of varying ethnicity and employment status, and whose children differed by gender, age and health condition.

Parents were recruited from the pool of families whose child had taken part in the CYP phase of the INSCHOOL project and had agreed to be approached for future associated research (Spencer et al. 2023a, 2023b).

To provide consistency with the CYP phase of the INSCHOOL project, the inclusion criteria were as follows:

- parents whose CYP participated in the INSCHOOL project
- parents with at least one child of secondary school age (11–18) with a LTC
- parents of children with a LTC from one of 11 clinics—allergies, asthma, chronic pain, colorectal surgery, cystic fibrosis, dermatology, diabetes, neuromuscular, oncology, rheumatology and sickle cell anaemia.

Parents were purposively sampled to include diversity by:

- Gender
- Employment status
- Ethnicity
- Time since diagnosis of child's health condition
- Children with an Education, Health and Care Plan (EHCP)
- Children with an Individual Health Plan (IHP)

An Education, Health and Care Plan (EHCP) is a plan for CYP in the United Kingdom who are aged up to 25 years and require more support than is available through a school's usual special education needs support. It is a statutory document. An Individual Health Plan (IHP) is a plan to set out a pupil's medical needs and care at school, including emergency arrangements and daily medical care. It can be used to support and plan for the impacts of medical needs on a child's education, inclusion and experiences in school. It is not a statutory document and can be used for any child with medical needs such as those with asthma or diabetes. Sampling to include a mixture of those with and without statutory and nonstatutory plans enabled us to capture a range of experiences.

Recruitment was by informed consent. Eligible parents were initially approached by a member of the research team known to them from the INSCHOOL study. Interested parents were followed up by the lead researcher for the parent study to further explain the research and provide an opportunity to ask questions. Recruited parents completed online consent and demographic forms.

### 3.3 Data Collection

Data collection methods comprised an interview phase and a workshop to inform, discuss and review emerging findings.

#### 3.3.1 Interview Phase

Interviews adopted a participant‐led approach and were designed to maximise opportunities for parents to participate. This included scheduling interviews around work or child‐rearing responsibilities and giving parents choice and control over the interview process. They could elect to be consulted in the manner that best suited them—face‐to‐face, online using Zoom or by telephone. They could choose to complete a preparatory activity (Figure S4) enabling them to consider any key issues they wanted to cover in advance. The rationale was that the preparation activity would give parents a chance to reflect on their child's school experiences and arrive at data collection with some control over topics introduced and prioritised. For parents who did not complete an activity, the intention was to retain a participant‐led dynamic by asking open questions reflecting those on the preparation activity and following the topics which parents chose to raise. A semistructured topic guide enabled targeting of key themes across parents if these had not been covered through the open questions (Figure S5). To develop an initial rapport, parents were invited to tell us what question they wanted to be asked. Space was afforded at the start of the interview for them to talk about their child and their child's medical condition.

It was recognised that parents might find interviews emotional. If this happened, they would be told they did not have to answer questions they were uncomfortable answering and could stop at any time. To ensure participants did not leave interviews distressed, established processes for sensitive interviewing were deployed. These included debriefing, assuring confidentiality and privacy, offering referrals and signposting to potential sources of support (Muraglia et al. 2020).

#### 3.3.2 Reflexivity Statement

All interviews were conducted by the main author, an established researcher with previous experience of working with parents in a school setting. This provided the researcher with a firm grounding to ask follow‐up questions and provide participants with a sense that the researcher understood their circumstances. Questions were open to allow participants to talk about the areas they wanted to cover, but the researcher used their experience and knowledge of the field to probe areas of support that parents may not have covered.

#### 3.3.3 Coanalysis Workshop

A participatory workshop was convened at the analysis stage. This involved parents who had provided interview data to cointerpret the findings. The workshop was conducted online, involving four parents of children of different ages and medical conditions and three facilitators. The workshop lasted 3 h and activities included (Figure S6):

- an overview of interview findings
- Activity 1—a verification of the child's needs—where participants highlighted which of the six key needs matched their experience and those that did not.
- Activity 2—a verification of the role parents play—where participants were given a list of phrases about the parent role that they could select as relating to their experience or not. They were then asked how undertaking these roles affected their relationship with school and their child.
- Activity 3—priorities for need. Participants were asked to signify three key needs that stood out as particularly important.

#### 3.3.4 Data Analysis

Interviews were audio recorded with consent and transcribed verbatim with any identifying information removed or anonymised at this stage. Interviews varied in duration from 30 to 180 min. Data were imported into NVivo software for coding and analysis.

A framework analysis approach was adopted (Ritchie and Lewis 2003), and the standard five steps were followed: data familiarisation, framework identification, indexing study data against the framework, charting to summarise the data indexed and mapping and interpretation of the patterns found within the matrices (Goldsmith 2021). The aim of this analysis was to provide additional depth and context to the school experiences described by CYP; therefore, the six needs identified in the CYP INSCHOOL project (Spencer et al. 2023a, 2023b) were used as the framework for analysing the parent data. An iterative approach was taken by the research team to assess the extent to which data mapped onto the existing INSCHOOL needs framework, discuss this fit within the team and go back to the original data to sense check these interpretations. All transcripts were coded by VH, with a 30% sample annotated and verified by MR. VH and MR met to discuss coding experiences. Discrepancies were resolved within the research team. Alongside assessing fit within the framework, the INSCHOOL research team (SP) remained open to the parent data enhancing or adding to the original needs framework and to refine what was included within each of the six needs.

To ensure the experiences and beliefs of parents were accurately reflected and minimise the possibility of misinterpretation, findings were verified with parents in the coanalysis workshop. The workshop afforded parents the opportunity to coproduce findings and help determine any recommendations to clarify and enhance the needs framework (Levac et al. 2010). Empowering patients and their parents to have control and influence in this way has previously been reported as beneficial to deepening understanding of health needs (Simpson 2006; Smith and Firth 2011). The workshop provided in‐depth discussion of emerging findings with parents who felt strongly that the findings presented reflected their views of and needs from the education system.

## 4 Results

### 4.1 Participant Details

The study sample comprised 27 parents (Table 1) of a child with a LTC who attended one of 10 clinics in a hospital in the North of England, UK. Recruitment targets for at least one parent from each clinical group and diversity of characteristics for the parent and child were broadly met. The achieved sample, however, was predominantly White British mothers with White British children, and it proved difficult to recruit a parent of a child with sickle cell anaemia.

**TABLE 1:** Participant characteristics of parents and their children with a long‐term physical health condition.

Baseline characteristic	*n*
Parent	
Mum	26
Dad	1
Gender	
Male	1
Female	26
Ethnicity	
White	21
Asian/British Asian	3
Mixed or multiple ethnic group	1
Prefer not to say	2
Work status	
Not currently working	4
Full‐time	16
Part‐time	7
Child age	
11–13	7
14–15	11
16–18	9
School year group	
Year 7	0
Year 8	3
Year 9	5
Year 10	8
Year 11	2
Year 12	3
Year 13	2
Left school	2
Prefer not to say	2
Health condition	
Allergies	2
Asthma	4
Chronic pain	3
Colorectal surgery	3
Cystic fibrosis	4
Dermatology	2
Diabetes	1
Neuromuscular	2
Oncology	3
Rheumatology	3
Duration of health condition	
1–3 years	4
4–6 years	2
7–10 years	4
11 years plus	17
Education, health and care plan	
Yes	4
No	20
Do not know	3
Individual health plan	
Yes	7
No	15
Do not know	5

### 4.2 Needs Analysis

Using the six fundamental needs framework from INSCHOOL (Spencer et al. 2023a, 2023b), parents' descriptions of their CYP's needs at school and parents' role in addressing them are described below and illustrated with interview extracts. Figure S7 provides an expanded quotes table.

#### 4.2.1 Need to Safely Manage My Health at School

Parents explained how their children's health conditions needed to be safely managed in school, but this need was not always adequately met.

There was a need for reasonable adjustments, safe administration of medication and treatment and to respond appropriately when CYP were feeling unwell. Examples included passes for time‐out, the lift or toilet and more lenient school rules.

We looked at how we can adapt things. Her blazer was annoying on her arm, we'd put a sleeve underneath, so she did not have contact with the blazer material. (Parent—14 years Chronic Pain)

Stupid uniform rules! She needs comfortable shoes, generally trainers. We had to fight with them. We had to explain what the situation was, how it affects her. (Parent—14 years Neuromuscular)

Although parents reported reasonable adjustments were commonplace, school practices for deployment were often a source of tension between school and family and were not consistently or sensitively applied. Parents spoke of children with colorectal conditions not being excused to use the toilet and accused of ‘messing about’. Some children with allergies were made to wear an armband signifying their allergy. This made children feel ‘disbelieved’, ‘labelled’ and ‘different’, which was perceived as ‘psychologically harming’.

What's the point of a toilet pass if he has to explain himself every time he needs to go to toilet in front of the whole class? (Parent—13 years Colorectal)

Parents explained that CYP needed access to empathetic, ‘medically trained’ staff. The need for supportive staff was central to parents being confident their child would be safe in school, and this was enhanced when a school nurse was available.

At high school, I've felt more at ease, knowing that there's actual fully registered nurses there. (Parent—15 years Allergies and Dermatology)

It was common for increasing responsibility to be given to CYP to manage their condition as they got older. This caused parental anxiety as they did not have an overview of whether medication was being taken or how effectively the child was managing health in school.

Primary school they had a plan that I could see … they needed a little bit more help with the Creon. Any medicines were recorded, but high school I haven't seen anything. (Parent—14 years Cystic Fibrosis)

Parents of children with allergies explained it was becoming difficult to obtain multiple EpiPens for staff, parents and children to help mitigate risk. Health messaging and advice delivered in schools for conditions such as diabetes, asthma or cystic fibrosis could be out of date, causing upset. Parents gave examples of children with type 1 diabetes being maligned for ‘eating too much sugar’, those with cystic fibrosis being given differential information about life expectancy that did not reflect advances in treatment and those with asthma being suggested breathing techniques which conflicted with consultant advice.

Policies to plan for medical needs emerged as inconsistent and there was often a reliance on parents to mitigate and manage risks from needs arising. Parent descriptions revealed little awareness of Individual Health Plans (IHP) suggested in government guidance for children with medical conditions in England (Department for Education 2015).

##### 4.2.1.1 Parents Compensate

The parent data showed that parents take an active role to ensure children's medical needs are met. Parents reported having to go into school to administer medication and make judgement calls about whether CYP needed to stay in school or receive emergency treatment.

##### 4.2.1.2 Parents Advocate

Parents also spoke of needing to advocate for children when needs were misunderstood or disbelieved.

They were not going to administer medicines because that wasn't the policy. You'd have to come in and give your child medicines but that's just not realistic. (Parent—14 years Cystic Fibrosis)

It was literally, you need to come and get her, because they did not know what to do. (Parent—17 years Chronic Pain)

Parents highlighted having to ‘battle’ or ‘fight’ to get appropriate support.

You were fighting against that policy … shoelaces were a big issue, getting changed. Eventually the school went, “okay, that's fine,” but you should not have to fight. (Parent—17 years Chronic Pain)

School's very difficult to deal with … it seems to be ill‐educated teachers, they just seem to put her in a box of “we do not know what to do with her” so that's it. (Parent—13 years Neuromuscular)

Confrontation with education providers had negative effects on parents' relationship with school.

#### 4.2.2 Need for a Flexible Education Pathway

Parent accounts showed a lack of compensatory support for education missed (due to ill‐health or medical appointments) or for learning with the limitations of their condition (e.g., fatigue, pain and reduced concentration). Overall, it was clear that catch‐up or keep‐up support was generally unsystematic and often left to the CYP, parent or a sole supportive teacher. There was little evidence of alternative education provision such as an online offer.

He did not have the skills to concentrate because he'd never acquired them. There were still some issues with brain fog and mental capacity as a result of his treatment. (Parent—18 years Oncology)

The downside is we cannot get any work provided. I think that's one of the failings that bothers me more now that she's in her GCSE years. (Parent—14 years Allergies and Dermatology)

Parent descriptions showed a need for flexibility in the education system. To make up for time missed, some had secured support by substituting lessons CYP were less able to participate in equally for greater input in core subjects.

Some parents highlighted the importance of not exerting too much educational pressure on CYP with LTCs. Not requiring children to attend full‐time, make up for lost learning, do homework or put pressure on themselves was what was felt to be required from the education system.

There was a sense amongst parents that CYP with LTCs were disadvantaged compared with peers. Their ability to achieve educationally was ‘despite’ what they received in school. Parents highlighted the need to ensure arrangements to address educational needs do not risk further stigmatising or penalising CYP.

He did really well … despite the school. (Parent—18 years Oncology)

It changed the path of her life. Her A‐levels were good and she got offered a place at Cambridge and had to do the Cambridge maths exam. She had a horrendous night. She would not let me write afterwards to say “actually this happened the night before” and therefore she did not get in. (Parent—19 years Diabetes)

Parents had mixed awareness of what CYP could be entitled to and varied experiences of securing effective support. Some were able to advise schools on what was available whilst others lacked awareness.

We were told by the school that if he's off sick do not contact the teachers for work … He was frustrated because he does not want to fall behind. (Parent—15 years Rheumatology)

When she sits exams, she gets 25% more time and can have breaks … [School] messed it up … I had to go in to sort it out. It was [her Dr] that said she was entitled to that not school. School did not have a clue. (Parent—17 years Chronic pain)

##### 4.2.2.1 Parents Compensate

Parents often made up for lost education by paying for private tuition. Some parents bore the cost of examination fees typically provided by state‐funded schools in the United Kingdom to enrol children for examinations as private candidates.

We used to pay for a private tutor for maths … because that's one of the subjects she struggled with. (Parent—17 years Chronic pain)

We paid for a private tutor, for six months, to help her try and catch up. (Parent—12 years Rheumatology)

##### 4.2.2.2 Parents Champion Equality

Several parents had obtained assistance for tests and examinations through reasonable adjustments, access arrangements and special consideration to adjust grades in reflection of illness.

##### 4.2.2.3 Parents Advocate

Overall, the evidence implied a need for greater clarity and consistency in what education support is offered when children miss school. The data showed that parents take on the burden of support for lost learning and educational opportunities.

#### 4.2.3 Need to Be Acknowledged and Listened to in the Right Way

Parent descriptions showed that school experiences were positively or negatively affected by the extent to which CYP felt accepted, listened to and able to control the narrative around their health. Parents explained that as the CYP progressed through school and became increasingly responsible for their health, they could become less keen to disclose aspects of health and how this impacted on education. CYP were increasingly keen not to ‘stand out’, ‘be different’ or be seen to be getting preferential treatment. Parents spoke of CYP concealing their condition from friends.

It was common for parents to report that their child had been subjected to unkind comments, unwanted attention or bullying. Children were ‘disbelieved’ or challenged about their condition and support requirements. Parents were clear that unwanted attention was harmful, upsetting and a source of tension. Parents felt schools did not do enough to intervene, and CYP did not want their parents to raise it as a concern for fear of victimisation.

We did not do anything because my son's obviously older, he does not want any fuss … He does not want to be thought of as the disabled kid who needs allowances. (Parent—15 years Rheumatology)

My daughter is in total denial there's anything wrong … She wants to blend in completely and knows that she needs things like the room on her own. (Parent—18 years Diabetes)

##### 4.2.3.1 Parents Compensate

Navigating their role in attending to their child's needs was challenging for parents. They grappled with the tension between ensuring schools were informed of their child's needs and the child's desire to control their own health. Balancing this affected parents' confidence that children were safe, with implications for parents' own emotional health.

There's part of you that feels like you are not doing enough, I should have tried harder and the other half of you is going, thank God we have managed to get through today. He's still here, that's all that matters. (Parent—18 years Oncology)

I do feel a bit to blame. I feel as if I should be doing more. I should be turning up at the school and saying, “Why have you not done this or that?” (Parent—14 years Rheumatology)

##### 4.2.3.2 Parents Advocate

It was important for the child and parent that CYP were listened to and treated in the way they wanted. Parents battled for children to be heard and protected. The evidence shows this negatively impacted on the school–home relationship. The role of the parent in advocating for the desire of their child to control the story of their health was difficult to navigate but deemed important due to resulting adverse effects on emotional well‐being across the family.

#### 4.2.4 Need to Be Included in and Supported by the School Community

Parent accounts showed it was important for CYP to feel they belonged at school and had supportive friends and school staff members. Making and sustaining friendships was difficult for some CYP. In other cases, parents described how friends were helpful.

My son's friends knew he carried an EpiPen. He showed them how to use it because that was going to save his life if he needed it. (Parent—16 years Asthma)

The presence of compassionate staff in school emerged as vital for CYP and parents. CYP valued having someone who they could rely on to fight their corner. Access to a consistent, reliable member of staff was important in fostering parental confidence and trust in the school. More negative experiences of school emerged from families feeling a lack of involvement in decisions about how their child's needs were managed.

She's well‐known to all staff. I think that gives her a sense of security. She can just go down there as many times a day as she wants to. So that's a big positive for me. (Parent—15 years Allergies and Dermatology)

I said … “[there's] bullying … that's why he has not been com[ing] to school. He's been so stressed … If anything happens … if his blood pressure goes sky high anything could happen to him … If it does, I'm gonna hold you accountable.” (Parent—13 years Colorectal)

Staff‐to‐staff communication was often highlighted as challenging. Parents were frustrated having to repeat information or secure support for arrangements already agreed. This was more apparent at secondary school where a greater number of staff members needed to be cognisant of the CYP's needs compared with primary school. Staff were described as more understanding when there were visible signs of the health condition. Invisible illness could result in staff not believing the CYP.

It doesn't change how you should teach my child, but you should be aware that something's happened in her past and still ongoing that you should be aware of … Clearly you don't. (Parent—16 years Colorectal)

It was important for CYP to be included in the school curriculum, activities and events in ways that accommodated their health condition whilst not making them ‘stand out’. Whilst some CYP were fully involved in school life, parental feedback showed that many felt marginalised. Multiple examples were given of CYP not taking part in physical education (PE), school trips, reward schemes or enrichment activities which caused parental frustration. When CYP were included, parents explained this was not always in ways that best accommodated their needs.

##### 4.2.4.1 Parents Compensate, Advise and Champion Equality

Feedback showed parents played a role in securing appropriate alternatives to enable their CYP to take part in activities and PE. They were also advising schools and championing inclusion.

She was made to get changed, and then just sat on the bench all lesson. I went in and said, “Look, either you involve her … if it's just keeping score or refereeing, or something …” and they were like, “Oh right, we'll do that” and then they came back and said, “You know what, we're having real difficulty, we don't know what to do.” (Parent—12 years Rheumatology)

Whilst parents wanted their child not to be excluded from all that school had to offer, they needed to feel confident their child's needs would be appropriately managed. Having a say in how needs were accommodated was important for parents.

#### 4.2.5 Need to Build Towards My Future

Parent accounts outlined the potential consequences of their CYP's health condition on future education plans and prospects.

Several explained how they were only starting to realise the full effects of illness or treatment the further their child progressed through school. Often it was when CYP reached key transition points that parents appreciated there were extenuating circumstances to be accounted for.

As she was getting older, it was becoming apparent how much the stroke was affecting her learning, and her education. (Parent—18 years Chronic Pain)

Transition points were challenging to navigate given CYP's increasing independence and parents felt more removed from the process. Often transfer of information was limited. When new schools took time to listen to parents and plan for the CYP's needs, parents felt more assured. Having a health professional visit to explain the CYP's heath condition was highly valued when offered but appeared more common in some health conditions than others. Parents whose child had moved onto university were particularly concerned medical needs were not being met.

The biggest shock is the lack of support between 16 and 18. Where is that support in education? Where's the understanding for those children leaving education, wanting to progress into apprenticeships, wanting to go to different educational providers, where's the support for them? (Parent—18 years Chronic Pain)

Attendance and how this was monitored by schools was a concern, with parents keen to ensure their child's attendance record did not penalise them from securing places at college or university as they got older. They also felt CYP's employment prospects were affected by experiences of school.

They're oversubscribed and attendance is a key factor on getting a successful application. (Parent—14 years Cystic Fibrosis)

The data showed that some parents ‘pre‐empted’ their child's future needs at an early stage whilst others had little awareness of issues which may arise, often until informed by health professionals or in retrospect.

##### 4.2.5.1 Parents Champion Equality

Parents explained they needed to ‘fight’ for schools to better manage attendance for pupils with LTCs and to secure needs were in place when children moved into new phases of education and beyond.

#### 4.2.6 Need to Develop Attitudes and Approaches to Help Me Cope in School

Parent narratives implied that CYP needed to develop attitudes and approaches to help them adjust to their circumstances and cope with day‐to‐day life in school.

Parents recounted examples demonstrating how CYP had positively triumphed despite obstacles.

She's handled it like a trooper … If she wasn't as strong as she is I think the let downs would have been catastrophic. (Parent—13 years Neuromuscular)

Additional narrative regarding children's capacity to cope emerged from the parent data. This was discussed with the INSCHOOL team and subsequently tested through revisiting the parent transcripts to explore similarities and complementarities for the existing needs framework. Several parental accounts showed that CYP had struggled to cope, with many experiencing anxiety, poor mental health and low self‐esteem. Examples were given of CYP avoiding school. Some parents explained CYP had directed anger towards teachers, authority figures or peers.

He's pretty robust and we'll never know whether he would have been this robust if he hadn't been through all of this. He saw it as a challenge, which sometimes led to bad behaviour, but also it meant that he was like “xxxx you, I'm going to do as well as I can”. (Parent—18 years Oncology)

Support for CYP's emotional well‐being and mental health emerged as notably lacking, with parents finding it difficult to negotiate support in school. Some had accommodated this through health professionals, private treatment, charities or familial support, but many were left waiting for support.

He was supposed to be put on the Mental Health list. I heard from them last week, they said they'd be in touch and that's the last I heard. Because they have got fed up of me and my very aggressive emails. (Parent—16 years Asthma)

She fell off the rails mentally, would not go out, get out of bed, cried all the time, she struggled with friendships, … she was … unkempt. She was just a state. (Parent—18 years Chronic Pain)

It was evident in the data that parents had endeavoured to foster positive attitudes to help CYP to cope, although some still reported CYP concealing their worries for fear of upsetting their parents.

He has got anxiety around being off school … because he doesn't want me to get into trouble. A child shouldn't have to worry about that. He has enough worries and stress. (Parent—15 years Asthma)

##### 4.2.6.1 Parents Advocate and Champion

As has been shown in previous needs, parents played a supportive role in ‘battling’ for support for unmet needs and for mental health input with consequent negative effects for the CYP, parent and home/school relationship. This was considered a ‘tricky balance’ for parents managing the instinct to protect and intervene, with the need to foster independence and capacity to self‐advocate.

You're trying to get them what they need … you get what they need and they do not use it. The amount of involvement we have had … As a teenager they do not want you. It's having to be there to fight for them in the early days and then hopefully letting them take over. (Parent—18 years Diabetes)

To lose control in high school … I understand there's a big difference between, talking to a teacher every day at primary school and not seeing anybody, but to lose that total control for me was extremely difficult. (Parent—14 years Asthma and Allergies)

Support to deal with the psychological and social effects of the condition was a key unmet need in parent narratives. Applying the existing needs analysis framework to the parent data has helped to identify and enhance the six needs of CYP by highlighting variation between the different stakeholder groups (Smith and Firth 2011).

**FIGURE 2:**
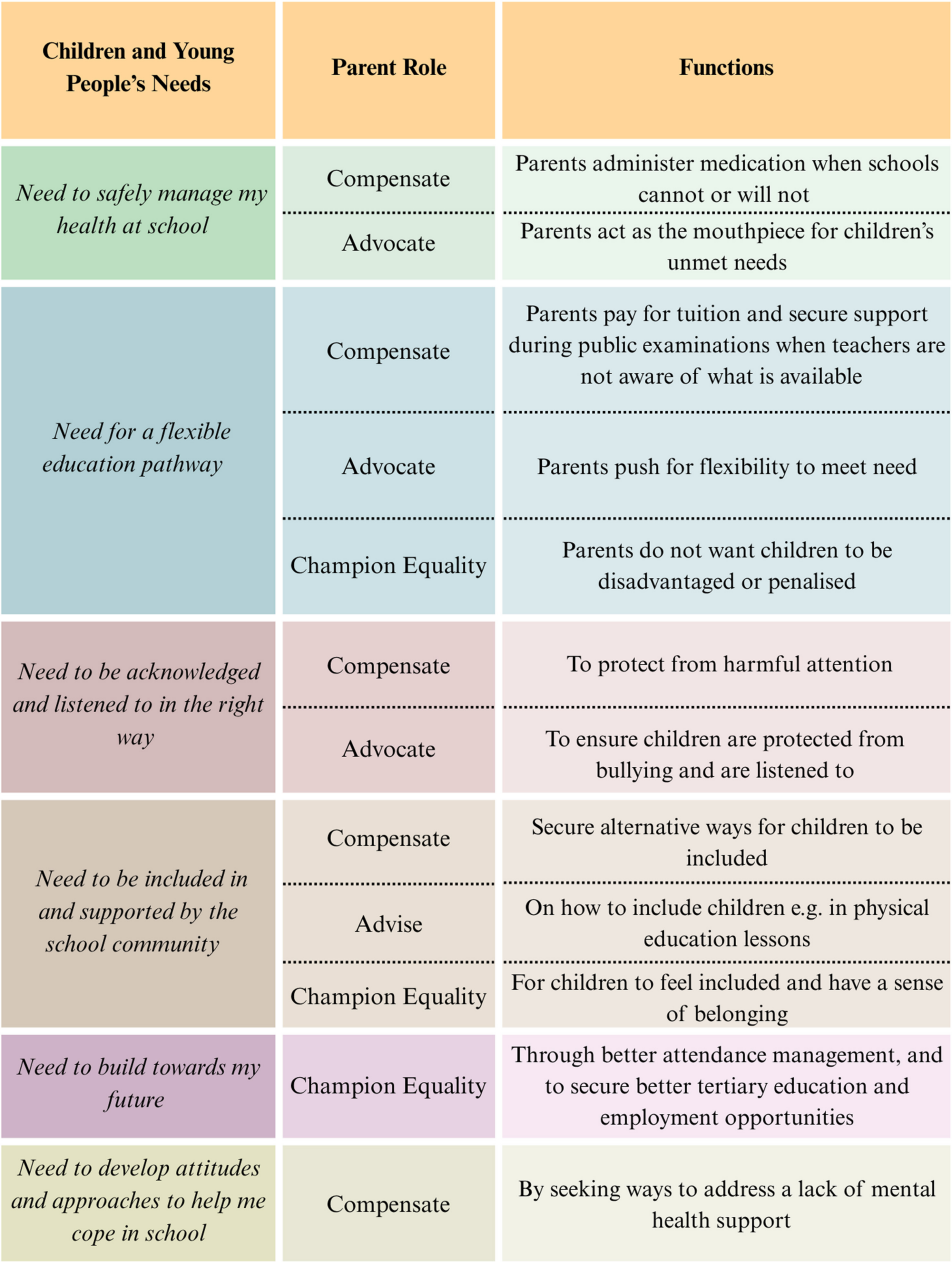
Summary parent role and functions in addressing CYP's needs at school.

**FIGURE 3:**
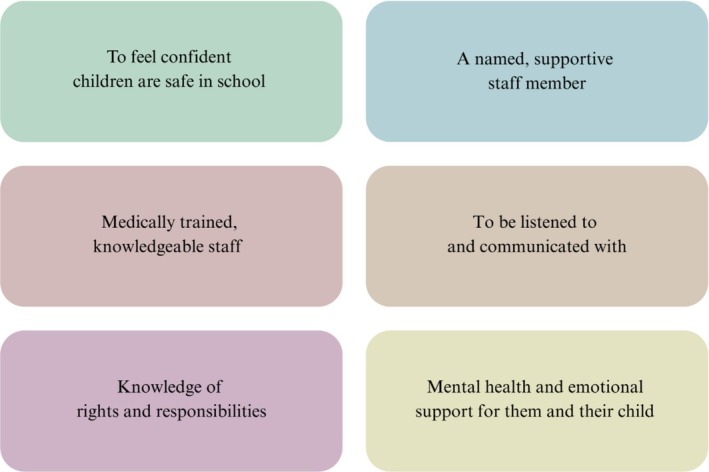
What parents want.

Overall, the data showed parents play four key functions to ensure CYP with medical conditions' needs are met (Figure 2). They compensate for limitations in support, advocate for CYP, advise schools on how to help and fight for equality and inclusion. Feedback showed parents want to feel confident their children are safe. They want to be listened to and informed of children's rights and the responsibilities of professionals to address these. They value having contactable, supportive staff who understand the condition. Mental health and emotional support for parents and children is a notable area of provision (Figure 3).

## 5 Discussion and Conclusion

### 5.1 Discussion

The parent project provides additional knowledge to that gathered from CYP with LTCs about school life (Spencer et al. 2023a, 2023b; Spencer et al. 2022). Whilst capturing the voice of CYP is essential in planning services designed for them (Bombard et al. 2018; Lea et al. 2018; Spencer et al. 2023a, 2023b; Taylor et al. 2018; Van Schelven et al. 2021), capturing parent voice helps to augment the data and provides a valuable additional perspective (Smith and Firth 2011).

We used an existing needs framework for categorising six needs of CYP with LTCs at school (Spencer et al. 2023a) and applied it to the parent data. Overall, the parent data corroborated the six needs from the INSCHOOL study (Spencer et al. 2023a, 2023b). CYP with LTCs have attendance and attainment needs, as well as requirements for medical, psychological and social support (Pini and Hopwood 2025; Spencer et al. 2022). They need to be included in and connected to the school community (Forrest et al. 2013; Hopwood et al. 2024; Knight et al. 2018; Lum et al. 2017; Pini, Hugh‐Jones et al. 2011; Pini, Gardner et al. 2016) and to be believed and listened to (Spencer et al. 2023a).

Adding to this, the parent voice enriched the data, providing more detail on the issues CYP face in relation to these needs. Our findings show there would be merit in broadening the definition of Need 6 in Spencer et al.'s framework (Spencer et al. 2023a) to better capture the emotional and mental health needs CYP experience and which the parent data reveals as absent. This supports evidence showing a greater incidence of anxiety and depression amongst children with life‐limiting conditions (Barker et al. 2023).

Across all areas of need, the parent data demonstrates the variety of roles parents play in negotiating support for CYP with LTCs at school. Resonant with previous studies, parents are taking on an advocacy role (Ewles et al. 2014; McCammon et al. 2018; Smith‐Young et al. 2022). As reflected in the literature, they act as mediators of provision (Ickmans et al. 2022), conduits for information (Bryan et al. 2021), brokers for health and education needs (Delloso et al. 2021; Lum et al. 2017; Pini, Hugh‐Jones, et al. 2011) and promoters of equity and inclusion (Hewitt‐Taylor 2009).

In addition, the data have shown how parents find it hard to balance an innate desire to support CYP's well‐being with the need to foster independence and reduce stigma, which has previously been reflected in research (Ickmans et al. 2022; McCammon et al. 2018; Palermo et al. 2014; Smith‐Young et al. 2022).

The parent accounts support existing evidence that parents can be passionate and ‘aggressive’ negotiators when acquiring provision (Smith‐Young et al. 2022). Securing support to meet CYP's unmet needs is shown in our data to lead to school conflict with consequent negative impacts for family‐school relationships and parents' own health and well‐being.

Our data shows how parents are compensating for gaps in the current education system for CYP with LTCs. They are meeting unmet needs by going into schools to deliver medication, paying for private tuition and mental health support, asking for access arrangements, advising staff on how to include CYP and ‘pushing’ for them to be listened to and their needs accommodated. The problem with this, as highlighted in previous research, is that it requires agency, capacity, knowledge, time and finances which not all parents have (Lee et al. 2021; Palermo et al. 2014; Smith et al. 2022; Smith‐Young et al. 2022) and thereby compounds known inequalities (Hopwood et al. 2024; Jay et al. 2023; Mon Williams et al. 2023; Spencer et al. 2023a).

The research data revealed some of the needs that arise for parents as a result of performing these functions. However, parental needs were not the focus of this study. Whilst there is some evidence (Devoy et al. 2022; Smith et al. 2022), there is a need for further research on the specific needs of parents and families and to better identify what constitutes effective needs assessment and intervention (Bradshaw et al. 2019; Gill et al. 2021).

The data have implications for healthcare and school professional practice. Children and young people would benefit from having a knowledgeable, named contact who can support them and their parents. Professionals should better assess CYP's needs at school and include them and their parents more thoroughly in the process of developing healthcare planning in school. They should provide information on CYP's rights and responsibilities for addressing them. Government guidance in England provided in 2015 on children with medical conditions at school would benefit from being reviewed and updated.

### 5.2 Strengths and Limitations

A key merit of this study was capturing the rich source of information residing with diverse parents across CYP with varying LTCs to complement and triangulate narratives from CYP. The participatory approach provided parents with the opportunity to positively influence research design and analysis. The voice of fathers was limited, and the connectedness of demographic data to themes emerging has not been fully explored. The research did not explore whether the children had other co‐occurring conditions that could have impacted their ability to navigate the school environment. Neither did it assess the influence of parental characteristics such as age, educational level, agency and advocacy on school experiences. Whilst the data have confirmed the needs CYP face, it has not fully explored the specific needs of parents and whether these are amplified by the activist role they play in navigating support.

### 5.3 Implications for Further Research

There is a need for further research to provide a more detailed understanding of parent needs in securing provision for CYP with LTCs and the impact on parents of ‘fighting’ for support. More investigation is needed to understand if the use of advised practices like the Individual Health Care Plan helps to better secure greater equitability of provision across the key areas of need identified.

### 5.4 Conclusion

This parent focused study augments an existing needs analysis for CYP with LTCs and adds to growing evidence that they have significant unmet needs in school and that these needs are shared across LTCs. Parents are compensating for failures in the system to meet these needs by plugging the gaps or advocating on behalf of their CYP.

The data highlights how the need for this parental role in navigating the school system can have negative implications for child and family relationships with school personnel. Navigating the system to secure support can have negative implications for home‐school relationships and parent well‐being. Requirements for parental agency to battle through health and education systems exacerbate health inequalities as not all parents are able to fulfil this function. Improvements are needed in the support currently offered to CYP with LTCs and their parents.

## Author Contributions

**Vicky Hopwood:** conceptualization (lead), methodology (lead), investigation (lead), formal analysis (lead), writing – original draft (lead), writing – review and editing (equal). **Simon Pini:** funding acquisition (lead), conceptualization (lead), methodology (equal), writing – review and editing (equal). **Megan Roker:** formal analysis (supporting), validation (supporting), writing – review and editing (supporting).

## Ethics Statement

Ethical approval was granted by East Midlands—Nottingham Research Ethics Committee in June 2023 REC Ref: 21/EM/0287; IRAS 302993.

## Consent

All participants in this study provided informed consent.

## Conflicts of Interest

The authors declare no conflicts of interest.

## Supporting information


**Figure S1** Six Common Needs of CYP with LTCs in school

**Figure S2.** Summary Parent Role and Functions in addressing CYP's needs at school

**Figure S3** What Parents Want

**Figure S4** Parent Preparation Activity

**Figure S5** Parent Topic Guide

**Figure S6** Parent Workshop Activities

**Figure S7** Sample Quotes

**Table S1** Participant Characteristics of Parents and their Children with a Long‐term Physical Health Condition

## Data Availability

Details of coding generated during the current study are available from the corresponding author on reasonable request. The data are not publicly available due to privacy or ethical restrictions.
