# Supplementary material for: Parents of Children and Young People With Long‐Term Physical Health Conditions—Experiences of Navigating School
Source: Child Care Health Dev. 2025 Jul 31;51(5):e70132. doi: 10.1111/cch.70132 (PMC12313002; doi:10.1111/cch.70132)
Supplement: Supplementary file 1 — Figure S1 Six Common Needs of CYP with LTCs in school [file CCH-51-e70132-s001.docx]

**Figure S1**

*Six Common Needs of CYP with long term physical health conditions (LTCs) in school.*


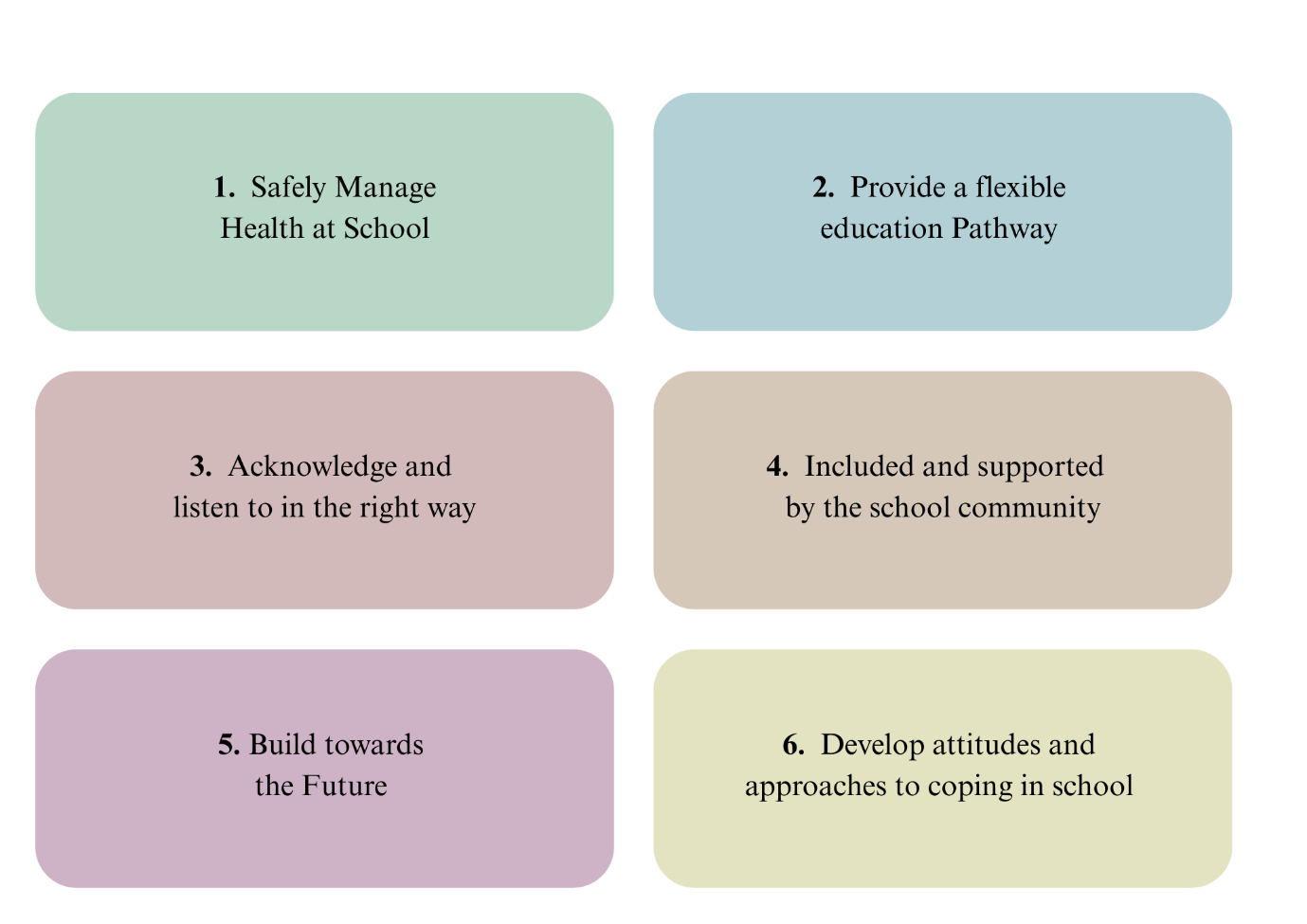


Note. CYP = Children and Young People.
