# Supplementary figures and images for: Parents of Children and Young People With Long‐Term Physical Health Conditions—Experiences of Navigating School
Source: Child Care Health Dev. 2025 Jul 31;51(5):e70132. doi: 10.1111/cch.70132 (PMC12313002; doi:10.1111/cch.70132)

**Figure S2**

*Summary Parent Role and Functions in addressing CYP’s need at school*


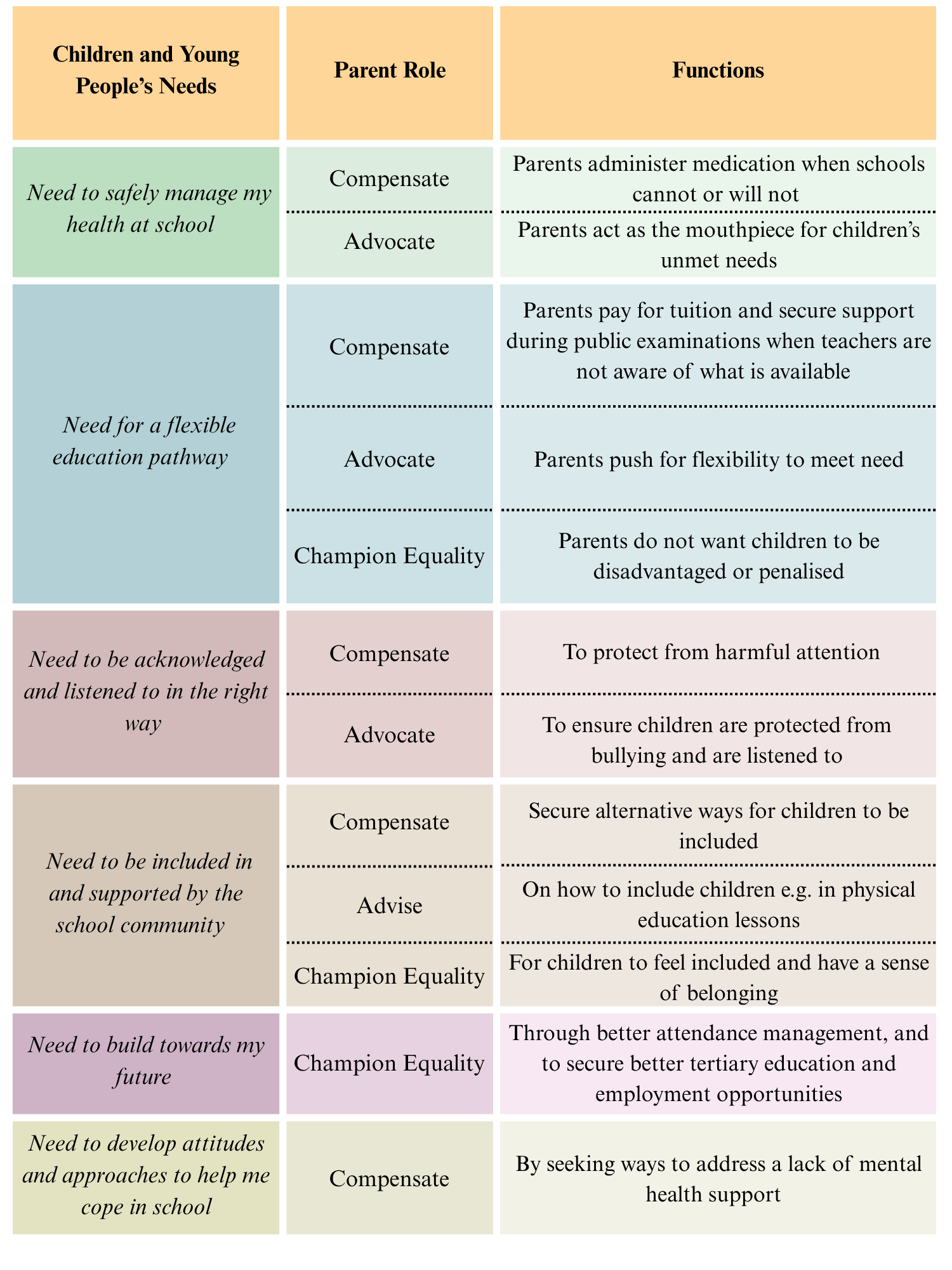

Supplement: Supplementary file 2 — Figure S2. Summary Parent Role and Functions in addressing CYP's needs at school [file CCH-51-e70132-s008.docx]

**Figure S3**

*What Parents Want*


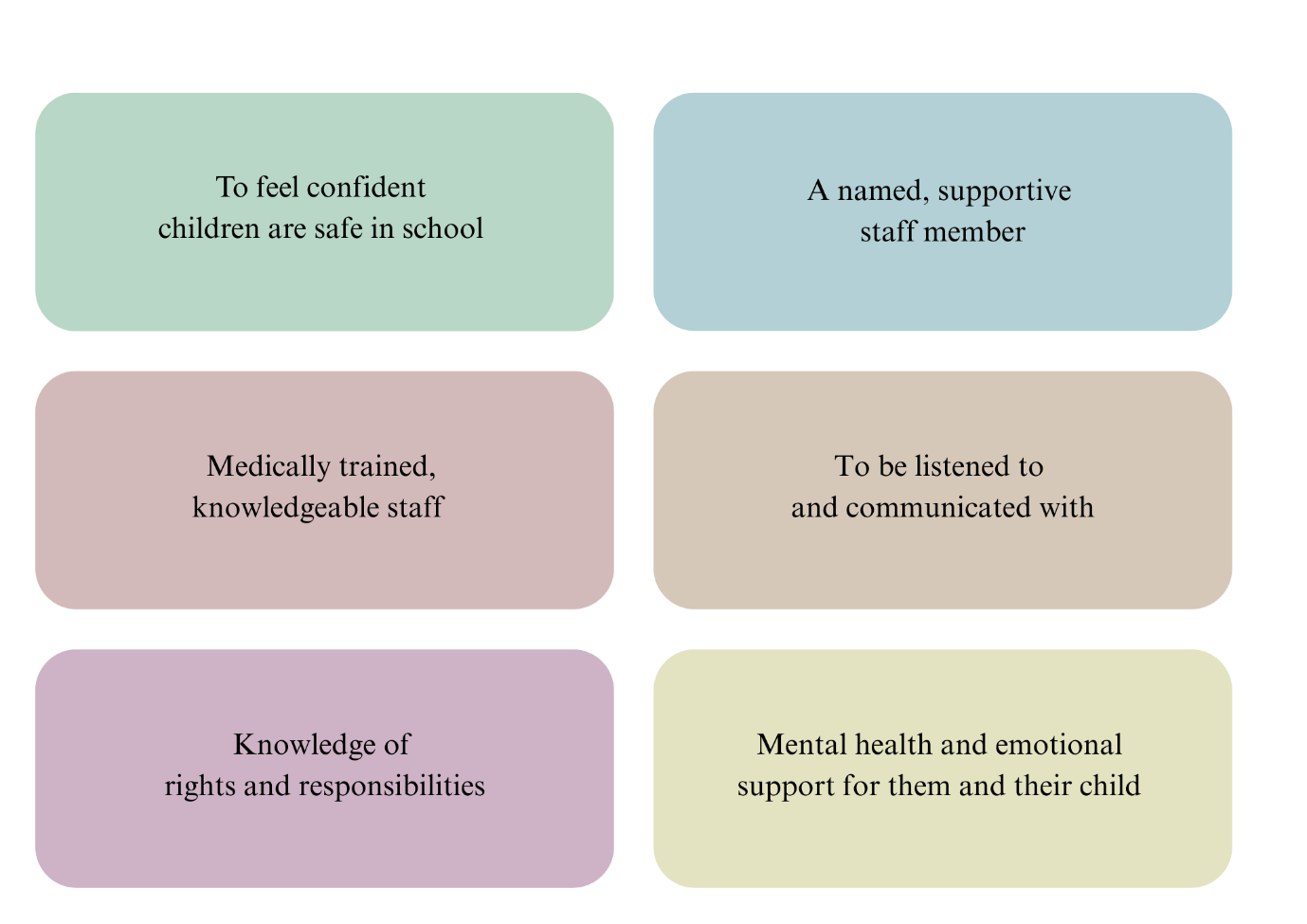

Supplement: Supplementary file 3 — Figure S3 What Parents Want [file CCH-51-e70132-s005.docx]

**Figure S6** *Parent Workshop Activities*

**Activity 1**

**Activity 2**

**Activity 3**


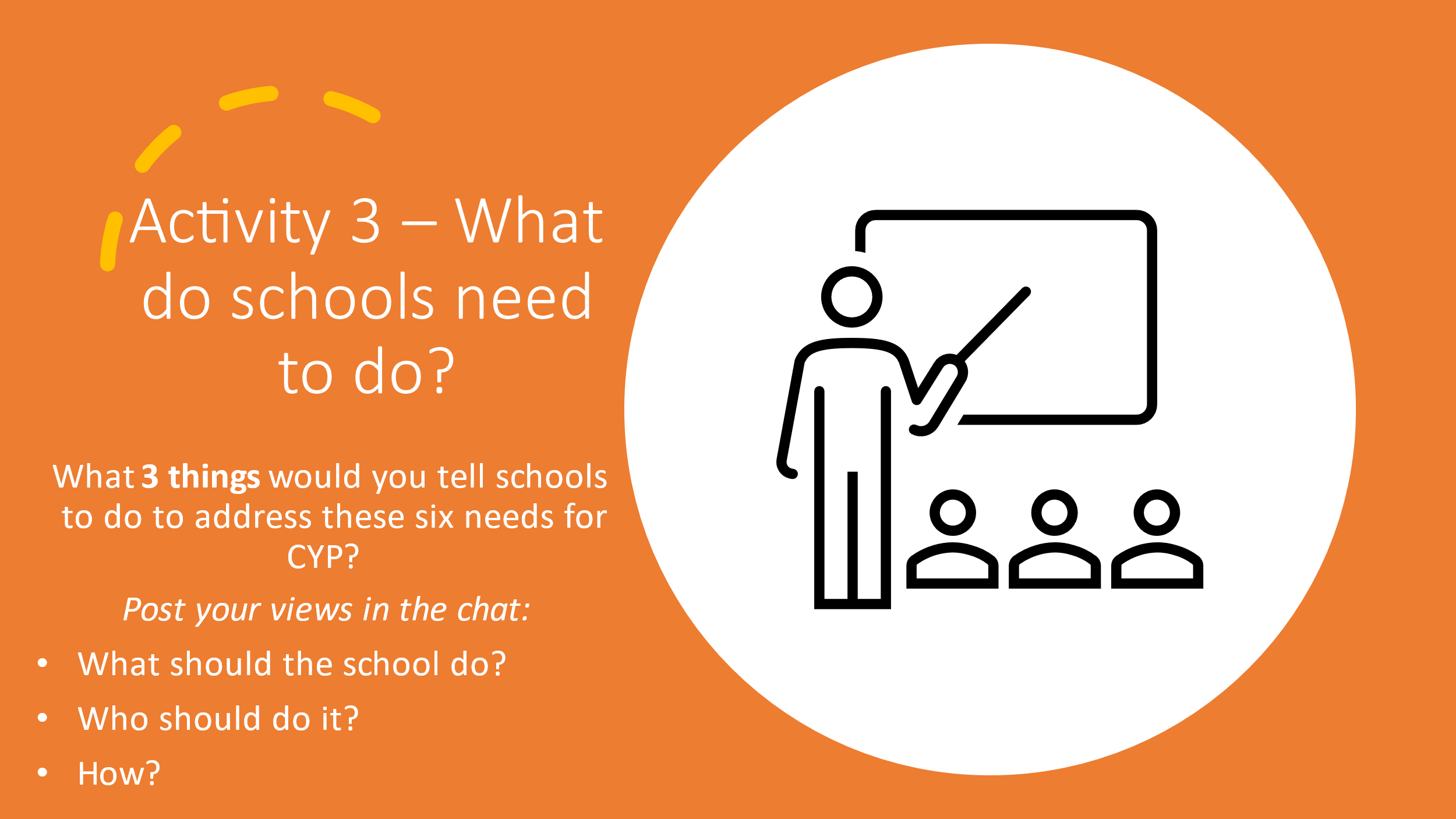

Supplement: Supplementary file 6 — Figure S6 Parent Workshop Activities [file CCH-51-e70132-s006.docx]
