# Supplementary material for: Parents of Children and Young People With Long‐Term Physical Health Conditions—Experiences of Navigating School
Source: Child Care Health Dev. 2025 Jul 31;51(5):e70132. doi: 10.1111/cch.70132 (PMC12313002; doi:10.1111/cch.70132)
Supplement: Supplementary file 4 — Figure S4 Parent Preparation Activity [file CCH-51-e70132-s003.docx]

**Figure S4 *Parent Preparation Activity***

1. Please think about up to three examples of **areas that have worked well at school** to help your child with their health condition.

| Jot down key things that have worked well at school: |
| --- |

1. Please think about up to three examples where you have, or your child has **encountered challenges** at school?

| Jot down key things where you have had challenges in relation to school: |
| --- |

1. Look at the table below which signify some key milestones in your child’s journey through school. Mark the milestone that has been most challenging for you to get support for at school.

| **Diagnosis** | **Returning or going back to school**  **(E.g. after or a period of treatment, or time off school)** | **Transition Points**  **(E.g. starting a new school, starting high school, leaving school, or moving into adult health services)** | **Important Exams or School Assessments** | **School Trips and Extra-Curricular Activities** |
| --- | --- | --- | --- | --- |
| **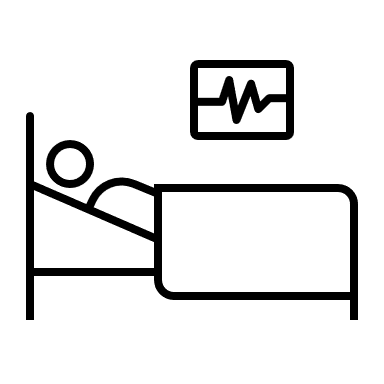** | **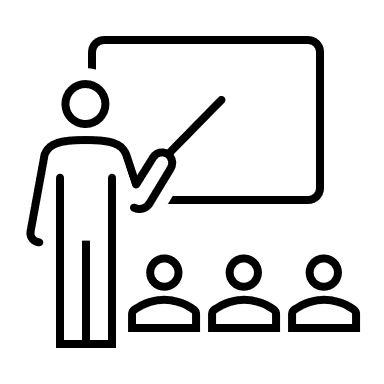** | **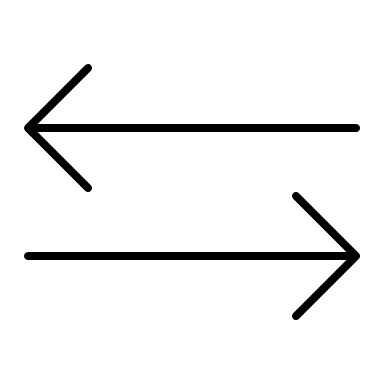** | **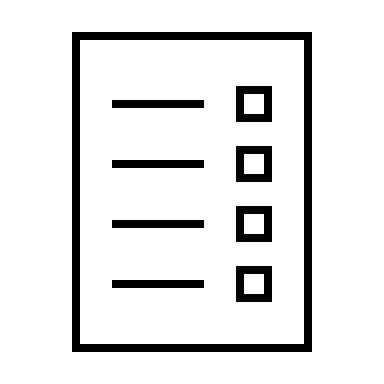** | **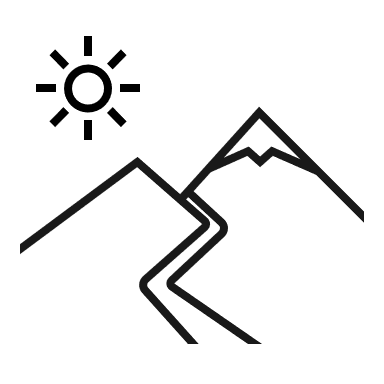** |

1. Use the box below to list any key words for things you want to remember to tell us about in relation to your child’s life at school?

| Jot down key things you want to remember to tell us about school: |
| --- |
