# Supplementary material for: Parents of Children and Young People With Long‐Term Physical Health Conditions—Experiences of Navigating School
Source: Child Care Health Dev. 2025 Jul 31;51(5):e70132. doi: 10.1111/cch.70132 (PMC12313002; doi:10.1111/cch.70132)
Supplement: Supplementary file 5 — Figure S5 Parent Topic Guide [file CCH-51-e70132-s004.docx]

**Background and Opening Questions**

1. Referring to survey - start by asking – “what’s the first question you wanted to be asked” and then proceed to asking them about their child’s condition – “what condition does your child have, how long they have had it, how often they come into the hospital for appointments, what treatment they need?”

# **Describe experience of school life - what’s worked and any challenges for the child in school.**

1. a. Refer to preparation activity – ask the participant to tell you if they completed the preparation activity and ask them to talk you through what they have completed.
2. Open question - Tell me about what school life has been like for your child? What areas have worked well for your child and what have you found to be more challenging?

# **Explore any issues relating to specific aspects of school life**

1. If not covered above – probe for detail or examples on:
2. **Attainment** - What if anything has the school done to help your child academically?
3. **Attendance** – How is attendance monitored and managed and have there been any issues around this?
4. **Inclusion** - How far has your child been included in school? To what extent are they being involved in all areas of school life (such as in PE, Trips, clubs)?
5. **Health** – How is your child’s health condition managed and supported in school?
6. **Psychological** – Does going through school/education raise any other issues for children with a long-term health condition (such as in relation to their mental health, well-being, psychological development)?

# **Exploring the issues for parents in navigating the system and securing support and help.**

1. As a parent of a child with a long-term health condition, tell me about your experience of liaising with education and health care professionals to meet your child’s needs in school and to get support for them?

**Exploring the effect and impact on families**

1. What’s the effect of all this on you as a parent and other members of the family?

# **Anything missed or to add**

1. Ask them to refer back to their preparation activity and ask them if there is something else they would like to add.
2. What advice would you give to other parents/families in the same position as you?

**Thank and Close**
