# Supplementary material for: Parents of Children and Young People With Long‐Term Physical Health Conditions—Experiences of Navigating School
Source: Child Care Health Dev. 2025 Jul 31;51(5):e70132. doi: 10.1111/cch.70132 (PMC12313002; doi:10.1111/cch.70132)
Supplement: Supplementary file 7 — Figure S7 Sample Quotes [file CCH-51-e70132-s007.docx]

**Figure S7** *Sample Quotes*

| **1 - Need to safely manage my health at school** | |
| --- | --- |
| Reasonable adjustments | *“They were very accommodating, they gave her a card so she could get out of lessons if she needed to, to go to the toilet.” – Parent of Child Age 16, Colorectal*  *“We looked at how we can adapt things. So, because her blazer was annoying on her arm, we'd put a sleeve underneath her blazer so she didn't have contact with the blazer material.” – Parent of Child Age 14, Chronic Pain*  *“He might have a kind of a buddy to help him, because obviously his mobility, he will find it difficult to carry his bag and things. So, school arranged for another child to be his buddy.” – Parent of Child Age 14, Oncology*  *“Stupid uniform rules! She needs comfortable shoes, generally trainers. We had to fight with them over that. We had to try and explain what the situation was, how it affects her.”– Parent of Child Age 14, Neuromuscular* |
| Negotiating adjustments/tension | *“Well, what’s the point of a toilet pass if he has to explain himself every time he needs to go to toilet in front of the whole class?” – Parent of Child Age 13, Colorectal*  *“She doesn’t do PE. In primary school the problem was that she just simply couldn’t participate, so she was just left to sit on the bench every lesson, just made to get changed into her PE kit and then just sat on the bench every lesson, and I kept going and saying, ‘Well get her to learn the rules and referee, or keep score’, ‘Oh right yes we can do that, we’ll do that’, nothing changed.” – Parent of Child Age 12, Rheumatology*  *“I was reassured that they would be able to cope with him before he started high school, nothing were a problem, he could have everything, there’s nurses if he’s not feeling well and there’s a room to go and have his private time and he’ll always be able to go to toilet and nothing were a problem but doesn’t seem to work out that way.” – Parent of Child Age 13, Colorectal*  *“I think if I didn’t trust [name] so much then I would have had to have gone in and start doing that stamping your feet, you will listen piece, which you just don’t want to do, do you because it just makes everything really, really difficult, you don’t want to be marked as the parent that’s an absolute nightmare.” – Parent of Child Age 14, Asthma and Allergies* |
| Importance/value of medically trained staff | *“I mean, as a parent, I say it won't come across so much as when a medical person points out what this child went through in terms of his treatment. So, I think it was very, very useful to have the Macmillan nurse there and, of course, the physiotherapist, to talk from his mobility point of view.” – Parent of Child Age 14, Oncology and Haematology*  *“At high school, I’ve felt a lot more at ease, knowing that there’s actual fully registered nurses there.” – Parent of Child Age 15, Allergies and Dermatology*  *“The Student Office is also the Medical Office so I would have thought that the staff, not necessarily have medical training, but even if they just had a sheet that said, ask these questions. Because sometimes they’ll ring me and go, ‘Oh, [name]’s got a stomach ache.’ And I just say, ‘Do you want to just put him on the phone’, because sometimes I feel like I’m talking to another child which is quite frustrating.” – Parent of Child Age 12, Colorectal*  *“That’s when I learned that staff wasn’t trained. They didn’t have, they obviously had basic first aid, but I feel there was, very much a, ‘oh, you’re alright, take your inhaler, catch your breath, you know, nothing’s wrong.’ And it’s like they didn’t know how to deal with it, they just thought, ‘oh, sit down for five minutes, you’ll be fine.’ And that’s obviously when the spasms start within the breathing and he gets worse. And then they’d ring me instead of an ambulance or they’d ring an ambulance and the ambulance would turn up and go, ‘well, did you give him an inhaler?’ And they’re like, ‘well, no, he didn’t know he needed it.’ – Parent of Child Age 16, Asthma* |
| Manages own health | *“Primary school they had a plan that I could see … they obviously needed a little bit more help with the Creon. Any medicines were recorded, but high school I haven't seen anything.” – Parent of Child Age 14, Cystic Fibrosis*  *“They are more confident themselves and more responsible for taking their own medicines, they’re still, you know, they’re still only young and need to be sort of, need that to be sort of checked on and, but I think the schools have done well with that.” – Parent of Child Age 14, Cystic Fibrosis*  *“She carries her medication with her everywhere …she carries it round school with her so that’s got her EpiPens in there, it’s got her inhalers, it’s got antihistamine, it’s got Paracetamol in there, basically it’s just got all the things that she needs in the event of an emergency. So in high school you’re allowed to carry that round which makes it better than primary school because in primary school you’re not and that was the one thing that always bothered me..” – Parent of Child Age 14, Asthma and Allergies* |
| School communications, policy, procedures and training | *“What I am saying is there should be some communication for a child who has chronic conditions and I’m sure people say the same thing for learning difficulties and all those other SLT stuff but there should be some process.” – Parent of Child Age 14, Asthma and Allergies*  *“All these developing things that could be centralised, I think would be extremely useful to school really. Because then as a parent you could add to it, trust that the school, you’re communicating like that.” – Parent of Child Age 12, Dermatology and Rheumatology* |
| Parental role – to be involved/relied upon to manage health at school | *“Yeah there were some things that were positive eventually but it was, they weren't going to administer medicines, things like that because they said that weren't the policy, if they were on antibiotics you'd have to come in and give your child medicines but obviously that's just, that weren't realistic for us.” – Parent of Child Age 14, Cystic Fibrosis*  *“They rang me to say that they’d rung an ambulance and I was to go to the school, directly, straightaway, to meet the ambulance to transfer him to the hospital. So I said, well I’m at [hospital] so by the time I get to you, the ambulance probably would have already, so we’ll miss, so I’ll meet him at [hospital]. Well they weren’t happy with that because that takes up a school teacher to take him.” – Parent of Child Age 14, Asthma and Allergies*  *“They just used to ring me up and come and get her, there was no ‘what can we give her’, like I’d take her medicine to school, they were able to give her Calpol and things, not that it made any difference because the pain was so extreme, but there was no let's just sit her down in a room with a teacher and let's see if she can do any work, it was literally, you need to come and get her, because they didn't know what to do.” – Parent of Child Age 17, Chronic Pain* |
| Parent role - battling for support for medical needs | *“You were fighting against that policy… shoelaces were a big issue, getting changed. Eventually the school went, okay, that's fine, but you shouldn't have to fight.” – Parent of Child Age 17, Chronic Pain*  *“School’s very difficult to deal with… it seems to be ill-educated teachers, they just seem to put her in a box of we don't know what to do with her so that's it.” – Parent of Child Age 13, Neuromuscular*  *“As he got into, like, Year 5 and Year 6, they wouldn’t allow him to have an inhaler on him because it was too much of a risk to other children. But, in my opinion, it was too much of a risk to his life to not have it on him. And that was a never-ending argument with them.” – Parent of Child Age 16, Asthma* |

| **2 - Need for a flexible education pathway** | |
| --- | --- |
| Keeping up/ catching up/concentration | *“He didn't have the skills to concentrate because he'd never acquired them. There were still some issues with brain fog and mental capacity as a result of his treatment.” – Parent of Child Age 18, Oncology*  *“The downside is we can’t get any work provided. I think that’s one of the failings and that bothers me more now that she’s in her GCSE years.” – Parent of Child Age 14, Allergies and Dermatology*  *“No, that’s up to them to chase that up, and so I think generally that it isn’t followed up! So they will miss out on information as a result of that. I don’t think there’s any process in place in the school to try to ensure that they catch up on stuff that they’ve missed, just their responsibility.” – Parent of Child Age 13, Cystic Fibrosis*  *“Trying to catch him up is very much down to us being really proactive and missing work and saying oh look, we’ll do it outside of school, because we don’t want him falling behind, rather than them saying how can we help keep him on track.” – Parent of Child Age 15, Rheumatology*  *“She'd miss school and then she'd have to then catch work up and ask friends regarding what work she'd missed, there was no contingency in place for her to catch up.” – Parent of Child Age 17, Chronic Pain*  *“There are times when she’s saying ‘I’m sort of drifting off, I can’t concentrate.’ And at the back of my mind I’m wondering, is it the medication she’s on, is it because of her condition?” – Parent of Child Age 14, Rheumatology* |
| Flexibility in education | *“So I said to them, ‘Well based on the evidence that we have on the neurological report, and the fact that we can prove that [name]’s brain is affected by numbers, her brain injury affects her learning as numbers and retention, can we not have the maths and put her on the health and social?’ They wouldn’t have any of it, no.” – Parent of Child Age 18, Chronic Pain*  *“So he approached me and said, ‘can we talk to school and ask them if I can do my core subjects and my computer sciences and when I have my other lessons, I can use those periods to catch up on work that I’ve missed from my core subjects?’ So I approached school and school said, ‘no, not until his attendance is better.’ [name] didn’t go back to school since May of this year, haven’t heard a word from them.” – Parent of Child Age 16, Asthma*  *“He’s on reduced timetables because he struggles with school anxieties.” Parent of Child Age 15, Asthma* |
| Exam adjustments and access arrangements | *“She’d be a separate room for reading but sometimes it works against you because you’ve got someone really distracting in the room and that did make her have a conversation and say ‘I realise the Tourette’s person’s got their own issues but that’s really, that’s putting everybody off’. So it’s, and it’s hard to manage but they do need it.” – Parent of Child Age 19, Diabetes*  *“For both his GCSEs and his A-levels, after a lot of effort we managed to get him extra time, but that was the SENCO sorting that out in secondary school and his Head of Sixth Form sorting that out for him in sixth form. That's all that they could do.” – Parent of Child Age 18, Oncology*  *“She also takes notes to school with that she might need additional help with exams, additional time potentially, laptop to use to do exams on so I'm pre-empting before we get to that point.” – Parent of Child Age 13, Neuromuscular* |
| Support for education lacking/unsystematic with mixed parental awareness of what’s available | *“We were at one point told by the school that if he’s off sick you don’t contact the teachers for work, he’s off sick meaning he’s not suitable to work, so he was a bit frustrated because obviously he doesn’t want to fall behind and he’s proactively seeking out to keep his education on track, but they said we don’t have to provide work because he’s off sick.” – Parent of Child Age 15, Rheumatology*  *“When she sits exams, she gets 25% more time and can have breaks … [School] messed it up… I had to go in to sort it out. It was [her Dr] that said she was entitled to that not school. School didn't have a clue.” – Parent of Child Age 17, Chronic pain* |
| Disadvantaged/achieve despite  Parent role to ensure not disadvantaged. | *“He did really well but that's despite the school.” – Parent of Child Age 18y, Oncology*  *“It changed the path of her life. Her A-levels were really good and she got offered a place at Cambridge and had to then do the Cambridge maths exam. She had a horrendous night. She wouldn’t let me write afterwards to say ‘actually this happened the night before and therefore didn’t get in’.” – Parent of Child Age 19, Diabetes* |
| Parent role - Private tuition/mitigation | *“At the end of Year 5 we paid for a private tutor, for six months, to help her try and catch up, because she was getting the highest grades for everything, she’s very, very clever, but obviously like I said her condition started, she deteriorated, she missed a lot of school, her standards dropped. So yeah we paid for a private tutor for six months, just to get somewhere where she should be, going into high school.” – Parent of Child Age 12, Rheumatology*  *“We used to pay for a private tutor for maths as well because that's one of the subjects she struggled with. On a Saturday morning we used to pay for her to go to a private tutor for a couple of years, to help with probably Year 9, 10 and 11.” – Parent of Child Age 17, Chronic Pain*  *“I emailed her SENCO, and I said, ‘Her learning isn’t working, it’s apparent that it’s not’. So I asked them to send the stuff in a PowerPoint, rather than [name] being part of the lesson, and we taught her in our own time.” – Parent of Child Age 18, Chronic Pain* |
| Parent role – to be aware of education support available. Rights and responsibilities. | *“GCSEs were messed up because school wasn't proactive enough, but her A-levels, everything's in place, hopefully, it's that not knowing what's out there.” – Parent of Child Age 17, Chronic Pain*  *“I wouldn’t say, they didn’t mention it initially but they were, it was her nurse at the hospital, her actual consultant who mentioned it and the nurse got in contact with school but they was more than happy.” – Parent of Child Age 15, Allergies and Dermatology* |

| **3 - Need to be acknowledged and listened to in the right way** | |
| --- | --- |
| Less likely to disclose with age/concealment | *“He didn't want the school to know in the first instance, he wanted a clean start, he didn't want the school to know about his medical history. I told them, and said he doesn't want you to know, but you do need to know, obviously.” – Parent of Child Age 18, Oncology*  *“We didn’t do anything because my son’s older, he doesn’t want any fuss raising because he doesn’t want to be thought of as the, the disabled kid who needs other allowances.” – Parent of Child Age 15, Rheumatology*  *“Option to go to a sixth form college and she decided not to partly because she didn't want to have to start explaining again about her background particularly and a sixth form college is very big. I mean she would have just been in three different classes and she would have had a tutor but she didn't want to have to start explaining again..” – Parent of Child Age 16, Colorectal*  *“The child doesn't want to be different, because [name] just wanted to blend in and not be different from anybody else.” – Parent of Child Age 17, Chronic Pain*  *“Children who just they’re okay with all the razzmatazz around medical conditions and they’re quite upfront about the fact that ‘I’m this so I get that, so I need this and I need that’, and then you’ve got my daughter who is in total denial that there’s anything wrong with her and wants to blend in completely.” – Parent of Child Age 19, Diabetes*  *“Not wanting to be seen to be different, she definitely has that with regards to what she wears at school and interestingly when we're on holiday she will wear clothes that, because she's got a lot of scarring on her legs and so when we're on holiday she doesn't mind people seeing that but at school and in day-to-day activities she doesn't want anybody to see.” – Parent of Child Age 16, Colorectal* |
| Comments and attention from peers | *“People ask her why she wears dressing on her hands and she’s like because I’ve got eczema. He’s like, you’re like a mummy! And she’ll like, usually laugh that one off. When she was newly in high school, she had people saying, oh you’re like a granny aren’t you, your skin’s so wrinkly and stuff like that impacted her a little bit.” – Parent of Child Age 14, Allergies and Dermatology*  *“Of course kids are cruel and he used to get into arguments with some lads because lads would say ‘oh do you have to wear nappies?’ and stuff like that.” – Parent of Child Age 13, Colorectal*  *“So in primary school, this girl had sat next to her, she'd then stood up in front of the whole class and shouted out, I cannot sit next to you,.., I'm going to have nightmares. So that was in front of thirty people.” – Parent of Child Age 17, Chronic Pain* |
| Disbelieved/ lack of knowledge by staff | *“He said ‘don’t do that, I’m immunosuppressed’, to which the teacher responded, ‘we don’t joke about things like that’, and he said, ‘no, genuinely, I’m immunosuppressed, I’m compromised because of the medication I’m on’, and she said, ‘it’s not fair for you to joke about things like that, people are going through cancers and things like that’ and she just wouldn’t believe him.” – Parent of Child Age 15, Rheumatology*  *“The mindset of the teachers that just don’t think he’s telling the truth when he has to go to the toilet. I’ve had these meetings, I was reassured that nothing would be a problem but obviously it’s becoming a problem.” – Parent of Child Age 13, Colorectal*  *“Student Services because they are continuously telling him that his technique is wrong and that when he has an asthma attack he needs to lie on the floor, flat, to help him with his asthma which obviously is none of the things that we’ve been advised to do with him.” – Parent of Child Age 14, Asthma and Allergies*  *“In the first two or three years, his Head, the Head Teacher asked him why he was wearing a cap, and he said because I've got no hair, and if the Head Teacher didn't know that there was child in his school in that situation so the Head Teacher's response was, ‘well, hurry up and get to class.’” – Parent of Child Age 18, Oncology* |
| Parent role intervene/support | *“She's just encountered all sorts of people commenting about her scar, commenting about her ability, commenting about how much school she's missed, things like that. This is one reason why I changed hours in my job, to be able to support and be there for her.” – Parent of Child Age 17, Chronic Pain*  *“So I had a meeting at school and said, ‘I don’t know what you’re playing at because you’re not looking after his wellbeing’ and they said ‘well we need’, I said ‘you had letters for when he first started from the consultant’, ‘it just said it’s his condition, doesn’t say anything’, ‘yes it does, it says that like at dinnertime, after his eating he could sit on the toilet for 20 minutes if he needs to go’, I said ‘so if you’re not going to let him go to the toilet then he don’t come to school, simple as.” – Parent of Child Age 13, Colorectal*  *“The detentions for absence, every week, I'm not joking, every week… Could you ring us and let us know if this is an authorised absence, so every week I would ring them back and say, hello, [name]’s got leukaemia, that's why he's absent.” – Parent of Child Age 18, Oncology* |
| Parent role – balance supporting child and empowering them to manage for themselves | *“We didn’t do anything because my son’s obviously older, he doesn’t want any fuss… He doesn’t want to be thought of as the disabled kid who needs allowances.” Parent – 15y Rheumatology*  *“My daughter is in total denial there’s anything wrong… She wants to blend in completely and knows that she needs things like the room on her own.” Parent – 18y Diabetes* |
| Parent role - in navigating needs has effect on parent | *“There's part of you that feels like you're not doing enough, I should have tried harder and the other half of you is going, thank God we've managed to get through today. He's still here, that's all that matters.” – Parent of Child Age 18, Oncology*  *“I do feel a bit to blame. I feel as if I should be doing more. I should be turning up at the school and saying you know, ‘Why have you not done this or why have you not done that?’” – Parent of Child Age 14, Rheumatology* |

| **4 - Need to be included in and supported by the school community** | |
| --- | --- |
| Friends and Peer support | *“So, all of my son’s friends, while he was at school, knew that he carried an EpiPen and he showed them how to use it because that was going to save his life if he needed it.” – Parent of Child Age 16, Asthma*  *“Children that’s grown up with him haven’t got a problem with it, it’s the ones that don’t that then say ‘well why is he having to keep going to toilet?’ and some of them say ‘well it’s not to do with you’, you know what I mean?” – Parent of Child Age 13, Colorectal*  *“I think she’s got a really good little support network going on there, and there’s no judgement, and she’s accepted for who she is.” – Parent of Child Age 12, Dermatology and Rheumatology* |
| Compassionate/ supportive staff | *“With the nurses, she’s well-known to all of them that. I think that gives her a sense of security. I think they always have her moisturiser creams in there so she doesn’t have to carry it round with her, she can just go down there as many times a day as she wants to, to re-moisturise. So that’s a big positive for me.” – Parent of Child Age 15, Allergies and Dermatology*  *“She’s well-known to all staff. I think that gives her a sense of security. She can just go down there as many times a day as she wants to, to re-moisturise. So that’s a big positive for me.” – Parent of Child Age 15, Allergies and Dermatology*  *“When she got a male TA as she went into Year 6, he was absolutely life changing in the education setting of primary school. He stood back, he observed her from a distance, he didn’t sit on her shoulder saying what she could and couldn’t do, and that actually made a massive difference to [name]. And it gave us the confidence as parents, when it was coming to high school.” – Parent of Child Age 18, Chronic Pain*  *“His form teacher was in touch a couple of times and she was fantastic and she stuck with him a bit through his whole school career, obviously he doesn't have the same form teacher, but she kept an eye on him. So she was great.” – Parent of Child Age 18, Oncology* |
| Staff Communication | *“The nurse did everything by the book in terms of physically ‘take that, here’s a sheet, do that’ to the other teachers, which I think if you didn’t have a nurse in school I think that would be really tricky that side of it and we never felt like she wasn’t included in anything.” – Parent of Child Age 19, Diabetes*  *“But I think that's just a problem with the secondary school system is the fact that because there's so many staff involved and from one year to the next you're moving from a group of ten teachers to a different group of ten teachers, you can't have the same conversation at the start of each year, it is on a computer system and that's staff's responsibility.” – Parent of Child Age 16, Colorectal*  *“Half the staff don’t even know there’s nothing wrong with [name] and so they ring me to complain about him. I’m like well, you do realise he suffers from cerebral palsy.” – Parent of Child Age 15, Asthma*  *“It doesn't change how you should teach my child but you should be aware that something's happened in her past and there are still ongoing things that you should be aware of and clearly you don't.” – Parent of Child Age 16, Colorectal* |
| Visible and ‘Invisible’ illness | *“It can change between day-to-day and some teachers are like well you did it last week so you can do it this week and my son’s one of those who will push himself continuously, he doesn’t want to look like the disabled child at school so he will push and quite often pushes too far, and even though the teachers are aware that he has this thing, they’re quite happy for him to push.” – Parent of Child Age 15, Rheumatology*  *“It doesn't change how you should teach my child particularly but you should be aware that something's happened in her past and actually there are ongoing things that you should be aware of and clearly you don't.” – Parent of Child Age 16, Colorectal* |
| Included/ excluded | *“Any school trips were dealt with really well, I was contacted beforehand to see what medication I’d be sending in terms of his needles, his Insulin and they would always take a back-up.” - Age 16, Diabetes*  *“I think I am quite lucky I've got a school which is quite understanding and they do welfare tests, they send a lot of cards and they collected some gifts for him like books and little puzzles.” – Parent of Child Age 14, Oncology and Haematology*  *“The staff were absolutely amazing and they said, ‘Yes, you can come into our room, what do you want us to tell your friends if they're asking where you are, what kind of thing do you want to say?’ and they would do anything to help.” – Parent of Child Age 16, Colorectal*  *“So she used to do some like refereeing, or write the scores down, or [name] asked if she could catch up on work, so that's when she'd do the maths and things like that, so she'd catch up on the odd bit of work and she'd just go and sit in reception.” – Parent of Child Age 17, Chronic Pain*  *“She could fall off, could hurt her spine, all these sort of things so yeah, school’s very difficult to deal with in that sense but it seems to be ill-educated teachers, they just seem to put her in a box of we don't know what the xxx to do with her so that's it.” – Parent of Child Age 13, Neuromuscular*  *“Or they might put her in a music lesson or something random with other kids and she’ll get the question then from the kids in the class, ‘have you been naughty for you to be sent in here?’ Fortunately [name]’s quite resilient in that aspect, she’s like ‘no, my skin's a bit bad, I can’t do PE so I’ve had to come in here.’ Sometimes they’ll just make her go stand outside and watch.” – Parent of Child Age 14, Allergies and Dermatology* |
| Parents role - mitigate | *“I basically went in and just said, ‘Look, either you involve her, she needs to use her wheelchair, so if it’s just keeping score or refereeing, or something’ and they were like, ‘Oh right, yeah, we’ll do that,’ and then they came back and said, ‘You know what, we’re having real difficulty, we just don’t know what to do.’” – Parent of Child Age 12, Rheumatology* |
| Parent role – advise | *“The teachers don’t really know anything about the condition and so just to have that brief chat with them was really helpful for them to understand a bit more about what my concerns might be or what special needs the girls might have really so that’s probably the most positive thing that’s been done at secondary school.” – Parent of Child Age 13, Cystic Fibrosis* |
| Parent tole - champion | *“I asked a teacher why [name] hadn’t been on the playground, they said, ‘Oh she can’t play out,’ and I was like, ‘Yes she can’ because she was walking by this point, alright she wasn’t great, but she was on her feet, and I said, ‘She needs to go and experience the playground, you can’t exclude her because you think she’s fragile.’” – Parent of Child Age 18, Chronic Pain* |

| **5 - Need to build toward my future** | |
| --- | --- |
| Understanding health implications on future | *“As she was getting older, it was becoming apparent how much the stroke was affecting her learning, and her education.” – Parent of Child Age 18, Chronic Pain*  *“And I said that, ‘I’m concerned that she can’t do numbers, and I’m concerned that she doesn’t retain the months of the year, I’m concerned that she can’t tell the time, I’m concerned that she can’t put a simple meal in the oven, and know what time it is. Aren’t you?’ And they were like, ‘Oh we haven’t really noticed.’” – Parent of Child Age 18, Chronic Pain*  *“I've got a friend who works in admissions at York, and she said, if you don't say anything and we look at this and go, he's only done two A-levels, he dropped the GCSE.” – Parent of Child Age 18, Oncology*  *“Because she’s got a learning disability as well as everything else, you know, she wasn’t learning at the rate of her peers, and as she was getting older in an education setting, it was becoming apparent how much the stroke was affecting her learning, and her education.” – Parent of Child Age 18, Chronic Pain*  *“I was concerned about [name] and numbers, particularly, and that the way things were at that moment in time, I was concerned for [name]’s future, and what that looked like for [name], going into adulthood.” – Parent of Child Age 18, Chronic Pain* |
| Transition points | *“I think for me the biggest shock is the lack of support between 16 and 18. Where is that support in education? Where’s the understanding, those children leaving education, wanting to progress into apprenticeships, wanting to go to different educational providers, where’s the support for them to succeed, and do their absolute best.” – Parent of Child Age 18, Chronic Pain*  *“Firstly it’s like nothing had ever transferred from primary to secondary. So her record of medication, her current position with her anxiety, anything like that, it was all brand new, and I don't understand that, because I kind of think well a child has a record, to their situation, surely that should be transitioning over.” – Parent of Child Age 12, Dermatology and Rheumatology*  *“I can’t fault them at all. And she wrote a letter to the secondary school just explaining the seriousness of it, which made it easier when we went for an open day and when she got through they were understanding.” – Parent of Child Age 18, Allergies* |
| Attendance | *“Then like obviously now the next couple of years going to be applying for college, and you could put in the notes and stuff like that when you apply to college that the attendances are this because I've got these appointments at the hospital every three months.” – Parent of Child Age 14, Cystic Fibrosis*  *“I know for a fact that they're sort of oversubscribed, and attendance is a key factor on getting a successful application.” – Parent of Child Age 14, Cystic Fibrosis* |
| Parent role – champion support and equality | *“The biggest shock is the lack of support between 16 and 18. Where is that support in education? Where’s the understanding for those children leaving education, wanting to progress into apprenticeships, wanting to go to different educational providers, where’s the support for them?” Parent – 18y Chronic Pain* |

| **6 - Need to develop attitudes and approaches to help me cope in school** | |
| --- | --- |
| Attitudes to accept and cope | *“She gets fed up of it, just constantly trying new medications and trying this and the side-effects. But she doesn’t often complain though, bless her.” – Parent of Child Age 15, Allergies and Dermatology*  *“I think [name]'s handled it like a trouper, I think if she wasn't as strong as she is the downs would have been catastrophic to be honest.” – Parent of Child Age 13, Neuromuscular*  *“[Name] is pretty robust and we'll never know whether he would have been this robust if he hadn't been through all of this or not, but he doesn't like being dealt with like this, and he saw it as a challenge, which sometimes led to bad behaviour, but also it meant that he was just like eff you I'm going to do as well as I can despite the school.” – Parent of Child Age 18, Oncology* |
| Triumph despite barriers | *“Even though she's still getting bullied, well it's stopped now, the grades have started to come upwards so it's testament to how well she manages to cope.” – Parent of Child Age 13, Neuromuscular*  *“She goes to parents evening and they talked about feedback and they’re like she’s had a lot of time off and we totally understand with her eczema but she never falls behind and she’s always there.” – Parent of Child Age 14, Allergies and Dermatology*  *“Obviously in the end he got an A and an A star.” – Parent of Child Age 18, Oncology* |
| Struggling/Negative coping strategies | *“He's pretty robust and he doesn't like being dealt with like this, and he saw it as a challenge, which sometimes led to bad behaviour, but also it meant that he was just like, xxx you, I'm going to do as well as I can.” – Parent of Child Age 18, Oncology*  *“He will not back down to the point like he’s thrown hand sanitiser across the classroom, he’s, kicked chairs, he just loses his temper. If he feels like he’s not being heard he will carry on.” – Parent of Child Age 15, Asthma*  *“I said ‘because he tells you how it is and so if he needs to go he goes’, so sometimes when one of his teachers said no he couldn’t go, it were like 10 minutes into lesson, he just got up and walked out.” – Parent of Child Age 13, Colorectal* |
| Anxiety/poor mental health/low self-esteem | *“I think she were more easy with it when she was younger and had less knowledge about it. As she’s getting older and she’s becoming more self-aware, you know, people looking at you, I think that’s had quite a bit of an effect on her mental health.” – Parent of Child Age 15, Allergies and Dermatology*  *“And we were asking her how it was, and she kept saying ‘It’s fine’ but it was clear that it wasn’t, and in the end we had our meltdown, she just crumbled and said, ‘I can’t do it.’ And when we asked, she couldn’t do what, she said, ‘All of it.’” – Parent of Child Age 18, Chronic Pain*  *“I said ‘he’s stopped eating now and he’s getting depressed with it.’” – Parent of Child Age 13, Colorectal*  *“[Name] fell off the rails mentally, wouldn’t go out, wouldn’t get out of bed, cried all the time, she struggled with friendship circles, she was just so unkempt, didn’t get a shower, oh just she was just a state by this point. And this went on for a good six months.” – Parent of Child Age 18, Chronic Pain*  *“He started questioning why it's happening to him.” – Parent of Child Age 14, Oncology and Haematology*  *“I mean she probably is fine, but sometimes having that neutral person to maybe have an option to talk to somebody, I think they can offload then a little bit as well, can’t they, any concerns or worries she might have. You just want them to be okay, don’t you?” – Parent of Child Age 14, Rheumatology*  *“Since her accident there has been a lot of anxiety with her. So I don't know if that then increased the pain level due to just people being horrible and children being horrible.” – Parent of Child Age 17, Chronic Pain* |
| Lack of/unsuitable mental health support | *“I’ve done things to make things safer around [name] when he was going through that stage. I binned all my knives, I got rid of everything that he could hurt himself with and nobody was interested to help me.” – Parent of Child Age 15, Asthma*  *“The counselling, they didn’t really, nothing came to light because it was after Covid and they sent me a leaflet and it was something at lunchtime where all the children if they have an issue they go and it’s like a group. But my daughter wouldn’t want to go, so she said you know, ‘In a group setting and everybody will know where I’m going’ and if she’s already feeling like different, yeah. So yeah, and there was nothing else really, that’s all that they have as a school.” – Parent of Child Age 14, Rheumatology*  *“Anxiety and bouts of depression, we’re still waiting, he’s been accepted by CAMHS, but that was, it’s over a year now, yeah…It came to a head because he’d started self-harming and we just had a really bad few days and so I rang the crisis line and then they forwarded the details as well and pushed for CAMHS referral and then I think we waited 6 months to get an assessment and then that was in February, I can’t remember, but I think that was in February… They’re stretched beyond belief..” .– Parent of Child Age 12, Colorectal*  *“But at her school there wasn't much there, she only ever had one and it was for about five to six sessions and it was like mindfulness things.” – Parent of Child Age 18, Chronic Pain* |
| Children protecting/ hiding issues from parents | *“He’s been really upset and he had a really hard time to the point that he wouldn’t really confide in me.” – Parent of Child Age 15, Rheumatology*  *“But now [name] has got anxiety around being off school when he’s poorly because he doesn’t want me to get into trouble and a child shouldn’t have to worry about stuff like that. He has enough worries and stress to live with.” – Parent of Child Age 15, Asthma*  *“He didn’t want to tell me because I was poorly, ‘I don’t want me mum worrying’. I says ‘you’ve got to tell me things that are happening because I can’t do anything about it if you don’t’ and I said ‘and you made yourself so poorly over this.’” – Parent of Child Age 13, Colorectal* |
| Parent role - fostering positive attitudes | *“It’s trying to make your own child feel comfortable and accept what’s going on and not to make them worried, always trying to be positive and, ‘get you sorted and you’ll be fine, something’s just not right’. So that’s all you can do really, and just hope for the best outcome of all.” – Parent of Child Age 13, Colorectal* |
| Parent role – fight | *“You’re trying to get them what they need… Finally, you get what they need and they don’t use it. The amount of involvement we’ve had to have… [then] as a teenager they don’t want you in their life. It’s having to be there to fight for them in the early days and then hopefully letting them take over. It all depends on what personality they’ve got as to how they deal with it.” – Parent of Child Age 18, Diabetes* |
| Parent role -“Tricky balance” | *“You’re trying to get them what they need… Finally, you get what they need and they don’t use it. The amount of involvement we’ve had to have… [then] as a teenager they don’t want you in their life. It’s having to be there to fight for them in the early days and then hopefully letting them take over. It all depends on what personality they’ve got as to how they deal with it.” Parent – 18y Diabetes*  *“To then lose control of him in high school and I know you do, I understand there’s a big difference between, you know, talking to a teacher every day at primary school and not seeing anybody, but to lose that total control for me was extremely difficult, really difficult and I’ve had to rein myself back in for not going in and knocking on doors and stamping my feet a bit.” Parent – 14y Asthma and Allergies* |
