# Supplementary material for: Parents of Children and Young People With Long‐Term Physical Health Conditions—Experiences of Navigating School
Source: Child Care Health Dev. 2025 Jul 31;51(5):e70132. doi: 10.1111/cch.70132 (PMC12313002; doi:10.1111/cch.70132)
Supplement: Supplementary file 8 — Table S1 Participant Characteristics of Parents and their Children with a Long‐term Physical Health Condition [file CCH-51-e70132-s002.docx]

**Table S1**

*Participant Characteristics of Parents and their Children with a Long-term Physical Health Condition*

| Baseline Characteristic | *n* |
| --- | --- |
| Parent |  |
| Mum | 26 |
| Dad | 1 |
| Gender |  |
| Male | 1 |
| Female | 26 |
| Ethnicity |  |
| White | 21 |
| Asian/British Asian | 3 |
| Mixed or multiple ethnic group | 1 |
| Prefer not to Say | 2 |
| Work Status |  |
| Not currently working | 4 |
| Full-time | 16 |
| Part-time | 7 |
| Child Age |  |
| 11-13 | 7 |
| 14-15 | 11 |
| 16-18 | 9 |
| School year group |  |
| Year 7 | 0 |
| Year 8 | 3 |
| Year 9 | 5 |
| Year 10 | 8 |
| Year 11 | 2 |
| Year 12 | 3 |
| Year 13 | 2 |
| Left school | 2 |
| Prefer not to say | 2 |
| Health Condition |  |
| Allergies | 2 |
| Asthma | 4 |
| Chronic Pain | 3 |
| Colorectal Surgery | 3 |
| Cystic Fibrosis | 4 |
| Dermatology | 2 |
| Diabetes | 1 |
| Neuromuscular | 2 |
| Oncology | 3 |
| Rheumatology | 3 |
| Duration of health condition |  |
| 1-3 years | 4 |
| 4-6 years | 2 |
| 7-10 years | 4 |
| 11 years plus | 17 |
| Education, Health and Care Plan |  |
| Yes | 4 |
| No | 20 |
| Don’t know | 3 |
| Individual Health Plan |  |
| Yes | 7 |
| No | 15 |
| Don’t know | 5 |
